# PIM kinase inhibitor, AZD1208, inhibits protein translation and induces autophagy in primary chronic lymphocytic leukemia cells

**DOI:** 10.18632/oncotarget.26876

**Published:** 2019-04-19

**Authors:** Fabiola Cervantes-Gomez, Christine M. Stellrecht, Mary L. Ayres, Michael J. Keating, William G. Wierda, Varsha Gandhi

**Affiliations:** ^1^ Department of Experimental Therapeutics, The University of Texas MD Anderson Cancer Center, Houston, TX, USA; ^2^ Department of Leukemia, The University of Texas MD Anderson Cancer Center, Houston, TX, USA; ^3^ Graduate School of Biomedical Sciences, University of Texas Health Science Center, Houston, TX, USA

**Keywords:** PIM kinase, chronic lymphocytic leukemia, protein translation, apoptosis, autophagy

## Abstract

The PIM1, PIM2, and PIM3 serine/threonine kinases play a role in the proliferation and survival of cancer cells. Mice lacking these three kinases were viable. Further, in human hematological malignancies, these proteins are overexpressed making them suitable targets. Several small molecule inhibitors against this enzyme were synthesized and tested. AZD1208, an orally available small-molecule drug, inhibits all three PIM kinases at a low nanomolar range. AZD1208 has been tested in clinical trials for patients with solid tumors and hematological malignancies, especially acute myelogenous leukemia. The present study evaluated the efficacy and biological actions of AZD1208 in chronic lymphocytic leukemia (CLL) cells. CLL cells had higher levels of PIM2 protein and mRNAs than did normal lymphocytes from healthy donors. Treatment of CLL lymphocytes with AZD1208 resulted in modest cell death, whereas practically no cytotoxicity was observed in healthy lymphocytes. To determine the mechanism by which AZD1208 inhibits PIM kinase function, we evaluated PIM kinase pathway and downstream substrates. Because peripheral blood CLL cells are replicationally quiescent, we analyzed substrates involved in apoptosis, transcription, and translation but not cell cycle targets. AZD1208 inhibited protein translation by decreasing phosphorylation levels of 4E-binding protein 1 (4E-BP1). AZD1208 induced autophagy in replicationally-quiescent CLL cells, which is consistent with protein translation inhibition. These data suggest that AZD1208 may elicit cytotoxicity in CLL cells through inhibiting translation and autophagy induction.

## INTRODUCTION

Identification of proviral integration site for Moloney murine leukemia virus-induced lymphoma resulted in discovery of a proto-oncogene, termed PIM which was transcriptionally activated due to this insertion [1]. The founding member was appropriately termed *PIM1* and the product of *PIM* is a Ser/Thr kinase that promotes tumor progression, transcription, translation, survival, and proliferation. After PIM-1, two additional isoforms of PIM kinases have been identified; PIM2 and PIM3 which are able to phosphorylate numerous substrates with regulatory functions in several cellular processes [2]. These kinases are constitutively active and are early responder genes to growth factors and cytokines. They are also highly conserved throughout evolution, yet *PIM-1, -2,* and *-3* triple-knockout mice are viable and fertile [3], providing a rationale that these kinases could be targeted in cancer.

PIM’s pivotal role for cancer in general and hematological malignancies in particular became apparent as these proteins are overexpressed in malignant cells. These kinases are required for the efficient proliferation of peripheral T lymphocytes [3] and are needed for Abelson murine leukemia viral oncogene–mediated transformation of pre-B cells [4] or Epstein-Barr virus infection [5]. These proteins are overexpressed in B-cell malignancies, including chronic lymphocytic leukemia (CLL) [6, 7], Burkitt lymphoma [8], chromosome 6 gain non-Hodgkin lymphoma [9], and mantle cell lymphoma (MCL) [10–12]. PIM kinases also exert their oncogenic effects through cooperation with other genes involved in B-cell malignancies, such as *c-MYC* [13], nuclear factor kappa B [14] and CD40 ligation [15]. Collectively these data elucidate the role of PIM kinases in B-cell malignancies and use of PIM kinase inhibitors for these neoplasms.

Because of the critical role of PIM kinases in hematological malignancies, several academic institutes and pharmaceutical companies developed PIM kinase inhibitors. This effort was further fueled by the elucidation of the PIM1 crystal structure [16]. The first two PIM kinase inhibitors were SGI-1776 [7] and Smi4a [17]. SGI-1776 inhibits all three PIM kinases at nanomolar range along with Flt3 and TrkA. However, owing to the formation of metabolites and toxicity found in early clinical trials, SGI-1776 was regarded as a nonviable clinical candidate. Smi4a is a 5-(3-Trifluoromethylbenzylidene) thiazolidine-2,4-dione that was identified by screening and more potently and collectively inhibits PIM1 than PIM2. Smi4a was tested in multiple cell types, including hematological malignancies [18, 19]. Several other 3,5-disubstituted indole derivatives were identified as PIM kinase inhibitors through high-throughput screening at Novartis [20]. Lead compound LGB321 is among the most potent pan-PIM kinase inhibitors, with Ki values of 1.0, 2.1, and 0.8 pM for PIM1, PIM2, and PIM3 kinases, respectively. Novartis’ clinical candidate, LGH447, is in clinical trials for patients with relapsed/refractory multiple myeloma (MM) (https://clinicaltrials.gov/ Identifier: NCT01456689) and acute myelogenous leukemia (AML, NCT02078609). AZD1208, developed by AstraZeneca, is a pan-PIM kinase inhibitor, with IC_50_ values of 0.4, 5, and 1.9 nM for PIM1, PIM2, and PIM3, respectively [21]. AZD1208 showed promising activity in acute myelogenous leukemia (AML) cell lines and primary AML blasts [21, 22] and was tested in a clinical trial (NCT01588548). Although it was well tolerated in a phase 1 clinical trial for patients with AML [21], due to modest activity in the clinic, AZD1208 is no longer in clinical development.

Our prior investigations in CLL cells demonstrated that SGI-1776 was cytotoxic for malignant CLL lymphocytes, and this cytotoxicity was associated with a decline in one of the early response genes (MCL-1) [7]. The decrease in MCL-1 levels occurred at both transcript and protein levels. In contrast, normal lymphocytes from healthy donors were spared from drug-induced cytotoxicity. SGI-1776 was also effective in AML [23], MCL [24, 25], and MM [26]. However, SGI-1776 targeted PIM kinases with different potency and induced different amounts of cytotoxicity in these hematological malignancies, suggesting that the action of SGI-1776 was context dependent.

On the basis of these data, we hypothesized that a pan-PIM kinase inhibitor such as AZD1208 would be effective in CLL cells. We investigated the biological and molecular effects of AZD1208 on primary CLL cells. Our study demonstrated that PIM2 protein and transcript levels were overexpressed in CLL and that AZD1208 induced cytotoxicity in CLL cells, but not in healthy lymphocytes. Protein translation was affected in CLL lymphocytes and there was an induction of autophagy after treatment with AZD1208.

## RESULTS

Most of the experiments were done with primary human CLL lymphocytes that were obtained from patients (*n* = 62) with the disease. Peripheral blood mononuclear cells were isolated from fresh blood samples obtained from patients. Previously, we have demonstrated that these samples contain 85 to 96% lymphocytes and 82 to 94% CD19-positive cells [27]. Normal lymphocytes were isolated from the peripheral blood obtained from healthy volunteer donors.

### Expression of PIM kinases and MCL-1 levels in primary CLL and normal lymphocytes

Expression of PIM1, PIM2, and PIM3 in CLL samples taken from four patients was compared with healthy lymphocytes taken from three donors (Supplementary Figure 1A). PIM1 expression was undetectable in the normal lymphocytes, and only one CLL sample expressed high levels of this protein. There were 2 bands observed for PIM2, and PIM2 and PIM3 expression levels were consistently higher in the CLL lymphocytes than in the healthy lymphocytes. Transcript levels for the three PIM kinases were also determined in CLL and healthy lymphocyte samples (Supplementary Figure 1B). *PIM1* and *PIM3* mRNA levels were similar in lymphocytes from healthy donors and CLL patient samples, but PIM3 levels were lower than PIM1 in both healthy donors and patients. However, PIM2 transcript levels were 4 to 9 times higher in CLL samples than in the healthy lymphocytes.

Antiapoptotic protein levels for MCL-1, BCL-X_L_, and BCL-2 were also determined in these samples (Supplementary Figure 1C) to validate our results in the CLL cells. In accordance with the molecular characteristics of CLL, the untreated CLL samples had higher levels of MCL-1, BCL-X_L_, and BCL-2 than did the untreated healthy lymphocyte samples. At the mRNA levels (Supplementary Figure 1D), MCL-1 and BCL-X_L_ transcripts were expressed at similar levels in both healthy and CLL samples; however, BCL-2 levels were 8 to 13 times higher in the CLL samples than in the healthy lymphocytes.

### AZD1208-induced cytotoxicity and effect of sera

Most kinase inhibitors bind to serum proteins and are sequestered from entering the cells. Binding is also dependent on the type of serum which affects overall results. To determine whether AZD1208 binds at a different specificity in commonly used sera, we performed cytotoxicity assays in samples obtained from seven patients with CLL. For this purpose, isolated leukemic lymphocytes were incubated in medium containing 10% fetal bovine serum (FBS) (Life Technologies), 10% human serum (Cambrex Biosciences), or 10% autologous serum (plasma isolated from the patients’ blood samples) (Supplementary Figure 2). In general, CLL cells cultured in FBS-supplemented media and treated with either 3-µM AZD1208 or 10-µM AZD1208 had higher rates of cell death than did CLL cells cultured in human or autologous media (after endogenous cell death was subtracted) in all the patient samples. CLL samples from four subjects showed a decrease in spontaneous cell death (after DMSO treatment) when supplemented with autologous plasma than when supplemented with FBS serum. Since these data would suggest that the type of serum affects AZD1208 cytotoxicity, cell death was normalized to endogenous cell death for each type of serum (not shown). The analyzed data show heterogeneity in AZD1208 cell death regardless of the type of serum used to culture samples taken from each patient. To maintain consistency, all further experiments were carried out using commercially available human serum.

### AZD1208-induced cytotoxicity

**Figure 1:**
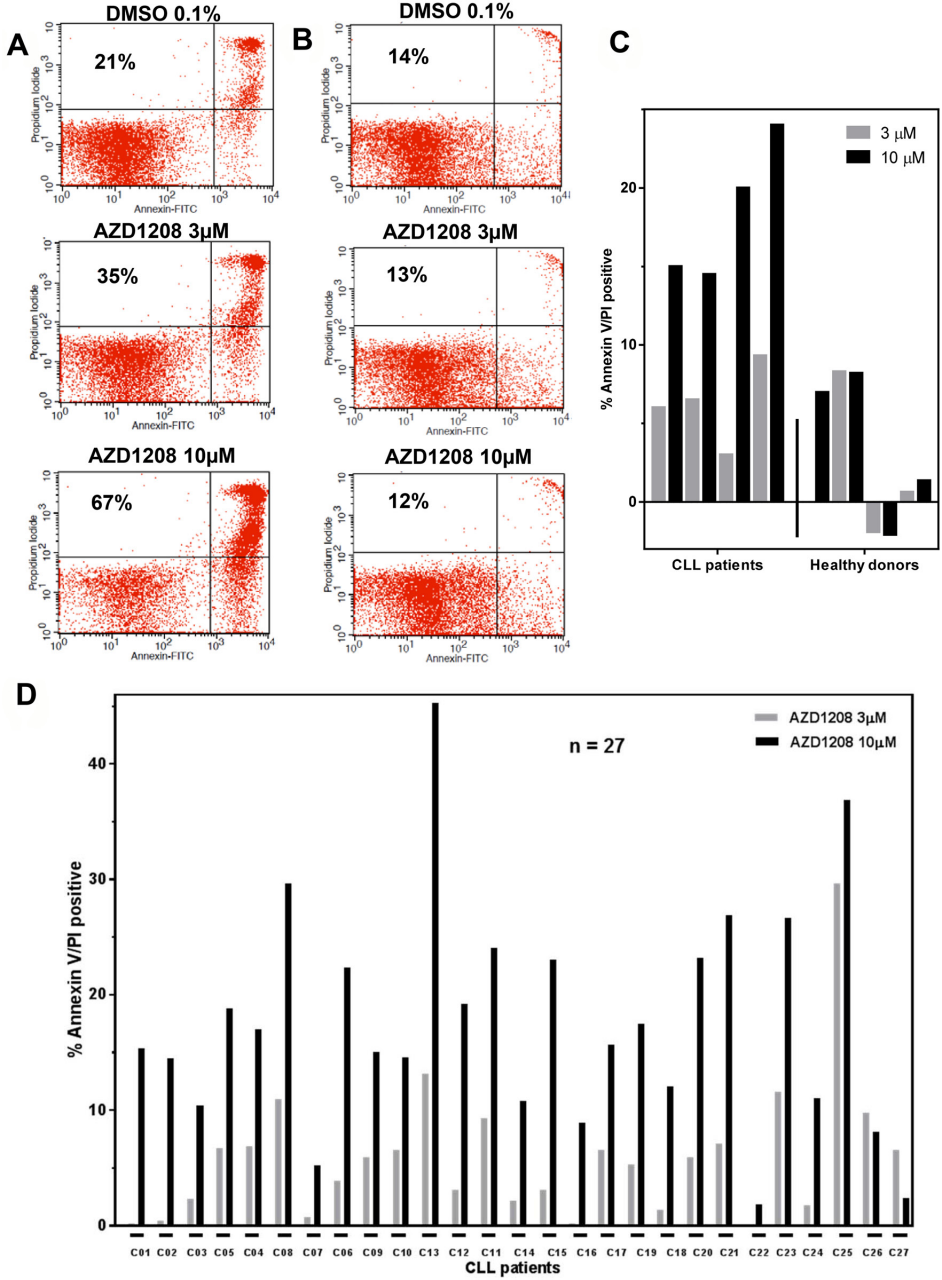
AZD1208-induced cell death Representative Annexin V and propidium iodide positive histogram data from a chronic lymphocytic leukemia (CLL) patient sample (**A**) and a sample from a healthy donor (**B**) after AZD1208 treatment. Malignant and healthy cells were incubated with dimethyl sulfoxide (DMSO), 3 µM, or 10 µM AZD1208 and evaluated for cell death by flow cytometry 24 h later. The number in the upper left quadrant represents percent cell death for that specific treatment. (**C**) Induction of AZD1208-mediated Annexin V/PI positivity in leukemia cells from four CLL patients and four healthy donor cells. Malignant and healthy lymphocytes were treated with DMSO, 3 μM or 10 μM AZD1208 for 24 h, stained with Annexin V/PI, and analyzed by flow cytometry. (**D**) Variability among CLL patient samples regarding AZD1208-induced apoptosis. Cells from 27 CLL patients were incubated with DMSO, 3 µM, or 10 µM AZD1208, and Annexin V/PI-positive cells were enumerated with flow cytometry.

Total cell death was determined as the sum of early apoptotic, late apoptotic, and necrotic cells after Annexin V/PI staining (Figure 1A). On the basis of the dose-response cytotoxicity results of AZD1208, 3-µM and 10-µM concentrations were selected for further experiments. The selected concentrations are clinically relevant based on an AZD1208 dose-escalation study in solid and hematological cancers [21] where Cmax ranging from 1,000 – 5,000 ng/mL were observed. This corresponds to 1.63 – 13 µM of AZD1208. Similar experiments were performed in cells from healthy donors. Figure 1B shows the representative nontabulated data for a healthy donor. The cytotoxic effect of AZD1208 was assessed in the same manner in four CLL samples and four healthy lymphocyte samples (Figure 1C). To determine the cytotoxicity resulting from AZD1208 treatment, we subtracted endogenous cell death from the cytotoxicity of each CLL sample. Samples from healthy donors treated with either AZD1208 concentration had an average 2% cell death rate, whereas CLL cells had a 6% cell death rate when treated with 3-µM AZD1208 and an 18% cell death rate when treated with 10-µM AZD1208. These data clearly suggest that AZD1208 causes negligible cytotoxicity in healthy peripheral blood lymphocytes and moderate cell death in malignant CLL lymphocytes.

To assess AZD1208-mediated cytotoxicity and heterogeneity in CLL, a total of 27 CLL patient samples were incubated with 3-µM AZD1208 and 10-µM AZD1208 for 24 h (Figure 1D). Endogenous cell death (median, 20%; range, 4%–50%) was subtracted from each CLL sample to compare the induction of cell death by AZD1208 treatment. When treated with 3-µM AZD1208, the 27 CLL samples had an average cell death of 6% ± 7; for those treated with 10-µM AZD1208, the average cell death was 18% ± 10. These results suggest that AZD1208 results in only modest cell death in CLL lymphocytes. Owing to this observation and to the fact that CLL cells do not proliferate without a supportive microenvironment, we evaluated the ability of AZD1208 to affect cell growth in MEC-1, a CLL cell line (Supplementary Figure 3). Our data show that MEC-1 cells treated with either AZD1208 concentration had less cell growth at all three time points compared to the MEC-1 cells treated with DMSO. At 48 hours, 3 µM AZD1208 and at 72 hours both 3 and 10 µM AZD1208 were significantly different than DMSO control. The cell death rate in MEC-1 cells treated with 10 µM AZD1208 ranged from 6% to 12% across all three time points.

### Impact of CLL prognostic markers on AZD1208-induced cytotoxicity

**Figure 2:**
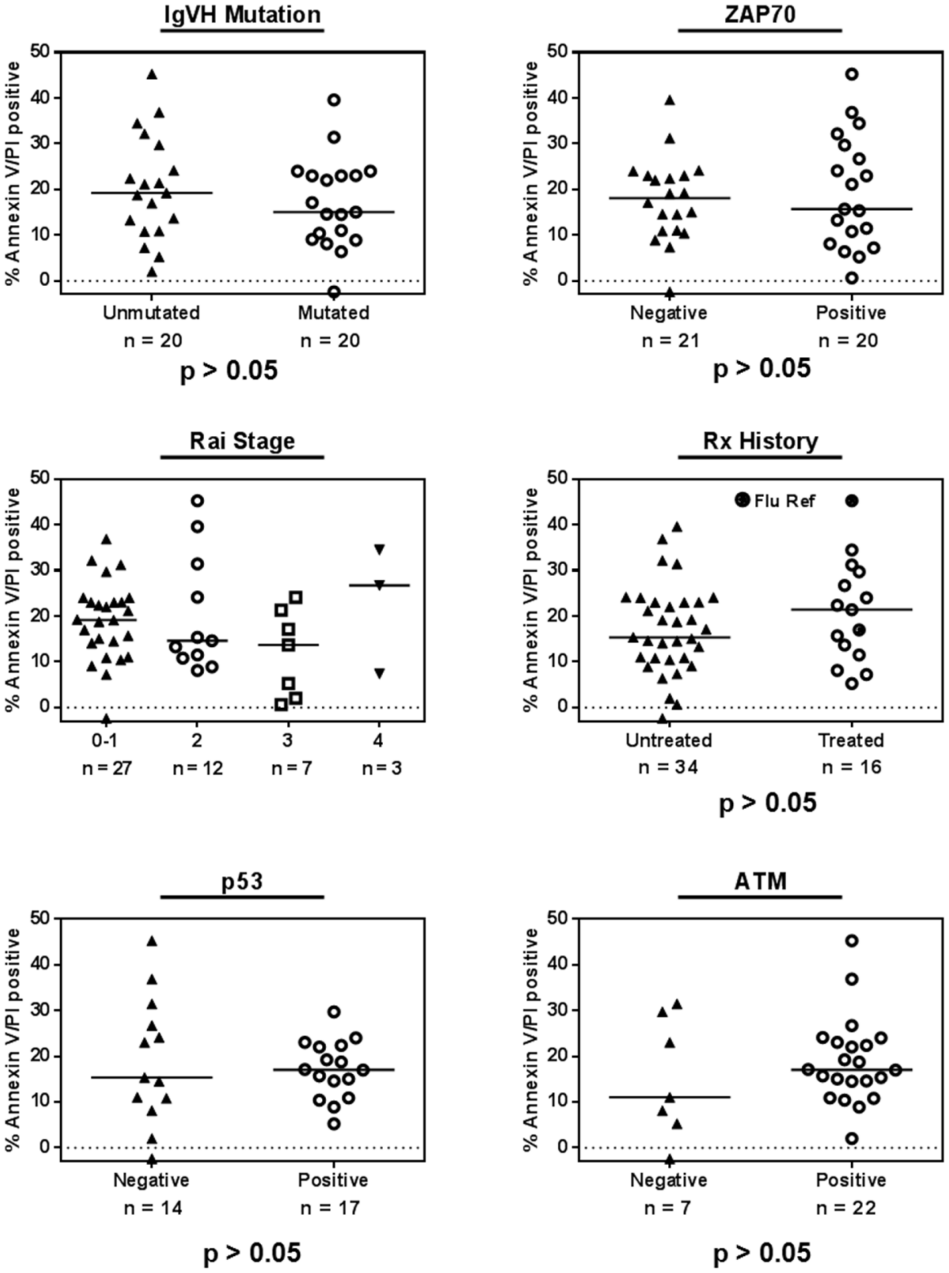
AZD1208-induced cell death and impact of chronic lymphocytic leukemia (CLL) prognostic markers CLL lymphocytes were treated with 10 µM AZD1208 for 24 h, and Annexin V/PI positivity was determined after the incubation period. The relationship between cytotoxicity and prognostic markers was plotted for immunoglobulin heavy-chain variable-region (IgVH) mutation, zeta-chain-associated protein kinase 70 (ZAP 70) status, Rai stage, previous treatment history, p53 (del17p), and ATM (del11q) status. The horizontal line in each graph represents the median for each group, and the *P* value (Mann-Whitney test) represents the analysis of each two sets per graph.

Because several cytogenetic and molecular markers have been implicated in predicting CLL sensitivity or resistance to chemotherapy or targeted agents, we analyzed the effect of these prognostic markers on patient samples that had been treated with 10-µM AZD1208 for 24 h (Figure 2). The immunoglobulin heavy-chain variable-region status was assessed in 40 CLL samples (unmutated = 20, mutated = 20) and statistical significance between the two groups was determined using the Mann-Whitney test. We also plotted cell death in CLL samples treated with 10-µM AZD1208 for 24 h according to molecular marker status: zeta-chain-associated protein kinase 70 (41samples; negative = 21, positive = 20), p53 (del17p, 31 samples; negative = 14, positive = 17), and ATM (del11q, 29 samples; negative = 7, positive = 22) status. We have compiled cell death according to previous treatment history (50 samples; never treated = 34, previously treated = 16, including two fludarabine-refractory samples) and clinical stage (Rai staging system). We did not find statistically significant differences for these prognostic factors, which suggest that all CLL samples were similarly sensitive to AZD1208 regardless of the patients’ prognostic marker status, clinical stage, or previous treatment.

### AZD1208 targets several PIM kinase substrates

**Figure 3:**
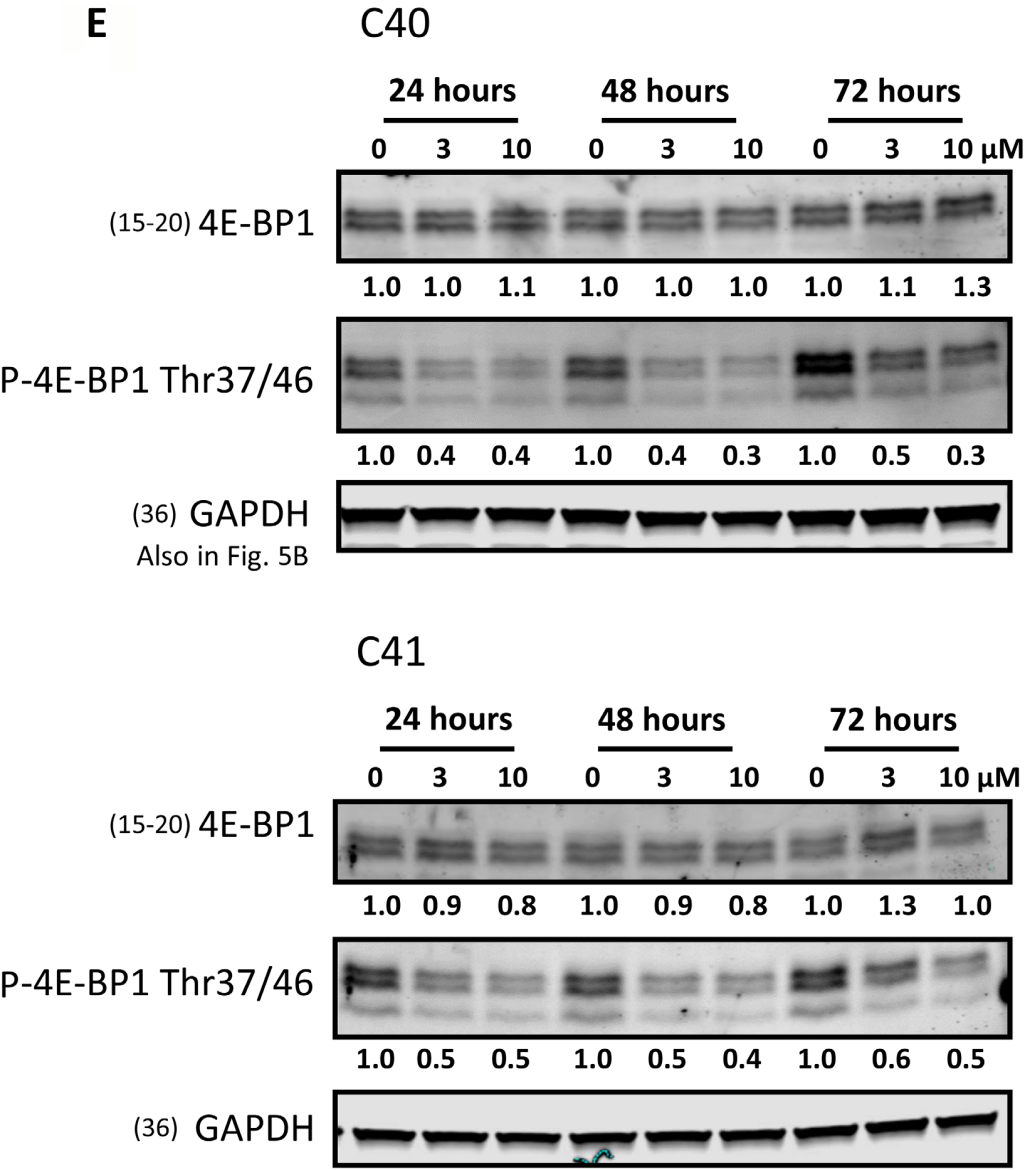
Effects of AZD1208 treatment on RNA and protein synthesis and PIM kinase substrates in chronic lymphocytic leukemia (CLL) lymphocytes (**A**) Uridine incorporation in the CLL lymphocytes. Cells were treated with dimethyl sulfoxide (DMSO) or indicated concentrations of AZD1208 for 23 h and then incubated with radioactive uridine for 1 h followed by radioactivity measured via scintillation counter. Data were normalized to cell number and were plotted as the percentage of untreated control. Treatments for each CLL sample were done in triplicate, and the bars represent the mean ± SEM. (**B**) Leucine incorporation in CLL lymphocytes. Cells were treated with DMSO or indicated concentrations of AZD1208 for 23 h and then incubated with radioactive leucine for 1 h followed by radioactivity measured via scintillation counter. Data were normalized to cell number and were plotted as the percentage of untreated control. Treatments for each CLL sample were done in triplicate, and the bars represent the mean ± SEM. (**C**) Effect of AZD1208 on phospho-c-MYC (Ser62), total c-MYC, phospho-histone H3 (Ser10), and total histone H3. CLL cells were treated with the vehicle DMSO or AZD1208 (3 μM or 10 μM) for 24 h; cells were then harvested, lysed, and analyzed via immunoblot. Phosphorylated-to-total protein ratios were calculated, and the numbers are depicted below the phosphorylated protein bands. (**D**) Effect of AZD1208 on phospho-p70S6K (Thr389), phospho-4E-BP1 (Ser65), phospho-4E-BP1 (Thr37/46), and total 4E-BP1 protein. Cells were treated with the vehicle DMSO or AZD1208 (3 μM or 10 μM) for 24 h; cells were then harvested, lysed, and analyzed via immunoblot. (**E**) Effect of AZD1208 treatment for 24, 48, and 72 h on phospho-4E-BP1 Thr37/46 and total 4E-BP1. Cells were treated with the vehicle DMSO or AZD1208 (3 μM or 10 μM) for 24, 48, and 72 h; cells were then harvested, lysed, and analyzed via immunoblot. Phosphorylated-to-glyceraldehyde 3-phosphate dehydrogenase (GAPDH) protein ratios were calculated, and the numbers are depicted below the phosphorylated protein bands. The membrane was probed with LI-COR imaging system using anti-rabbit and anti-mouse with distinct fluorescent wavelengths. When more than two proteins with the same molecular weight needed to be detected, the membrane was gently stripped and re-probed. In such cases, loading control remains same. Note: GAPDH loading control for Figure 3C, 3D (bottom gel) and Supplementary Figure 4A is the same. Note: GAPDH loading control in Figures 3E and 5B is the same.

PIM kinases have been implicated in phosphorylating several proteins at serine or threonine sites. These modifications are implicated in transcription, translation, apoptosis, and the cell cycle. Because CLL cells obtained from peripheral blood are replicationally quiescent, we did not evaluate AZD1208’s effect on cell-cycle proteins targeted by PIM kinases. Seven CLL samples evaluated for inhibition of global RNA synthesis with 3 and 10 µM AZD1208 showed heterogeneity in response compared to DMSO alone (Figure 3A). CLL samples (*n* = 7) treated with 3 and 10 µM AZD1208 had an average decrease in RNA synthesis of 26% (range, 0–55%), and 25% (range, 0–60%), respectively. This change was significantly different than the control value (*p* = 0.0003 and 0.001 at 3 and 10 µM, respectively). Similarly, CLL cells treated with 3 and 10 µM AZD1208 had an average decrease in protein synthesis of 22% (range, 3%–33%), and 43% (range, 14%–65%), compared with the control (not shown). To further assess, impact on protein synthesis, 11 patient samples were tested (Figure 3B) which showed heterogeneity yet significant decrease (*p* = 0.0001) in protein synthesis.

Because five of the seven CLL samples showed a decrease in global RNA synthesis, we used immunoblot analysis to determine the protein levels of c-MYC and histone H3, which are substrates of PIM kinases, in five CLL samples (Figure 3C). AZD1208-treated samples, regardless of concentration, did not have a significant change in the phosphorylation status of c-MYC at Ser62 or histone H3 at Ser10.

Because some AZD1208-treated CLL samples had lower global protein synthesis, we performed immunoblots to assess the phosphorylation levels of several PIM kinase protein translation targets, including the translation repressor protein 4E-BP1, phospho-4E-BP1 Thr37/46 and Ser65, and the phosphorylation status of p70S6K, the kinase that regulates translation (Figure 3D). AZD1208-treated samples had significantly lower phosphorylation levels for 4E-BP1 Thr37/46 than the control (*P* = 0.002). Hypophosphorylated 4E-BP1 interacts strongly with the translation initiation factor eIF4E, whereas hyperphosphorylated 4E-BP1 releases eIF4E. Phosphorylation of 4E-BP1 occurs sequentially: phosphorylation at Thr37/Thr 46 is followed by Thr70 phosphorylation, and the amino acid residue Ser65 is phosphorylated last. Our data show that AZD1208-treated samples had significantly higher phosphorylation levels of 4E-BP1 at Ser65 than did the DMSO control (*P* = 0.001). Due to the observed decrease in 4E-BP1 phosphorylation levels at 24 h, additional immunoblots were performed on two patient samples treated with AZD1208 up to 72 h (Figure 3E). Phosphorylation levels for 4E-BP1 Thr37/46 were decreased by 50% and maintained at that level compared to vehicle control when treated with 3 or 10 µM AZD1208 for 24, 48, and 72 h. Together these results may suggest that the hypophosphorylated status of 4E-BP1 could be attributed to the PIM kinase inhibitor blocking further phosphorylation of the repressor protein resulting in translation repression by not allowing release of the initiation factor eIF4E.

### Effect of PIM kinases on survival proteins in CLL and on inhibition by AZD1208

In previous studies, we found that treating CLL cells with the PIM kinase inhibitor SGI-1776 inhibited downstream PIM kinase substrates and affected the MCL-1 protein, which may cause drug-induced apoptosis [7]. To test this interaction in the current study, we used immunoblot analysis to determine MCL-1, BCL-2, and BCL-X_L_ protein levels in our CLL samples (Supplementary Figure 4A and 4B). AZD1208-treated samples showed no consistent and significant change in these three antiapoptotic protein levels. There was limited PARP protein cleavage.

### Induction of autophagy in CLL cells by AZD1208 treatment

**Figure 4:**
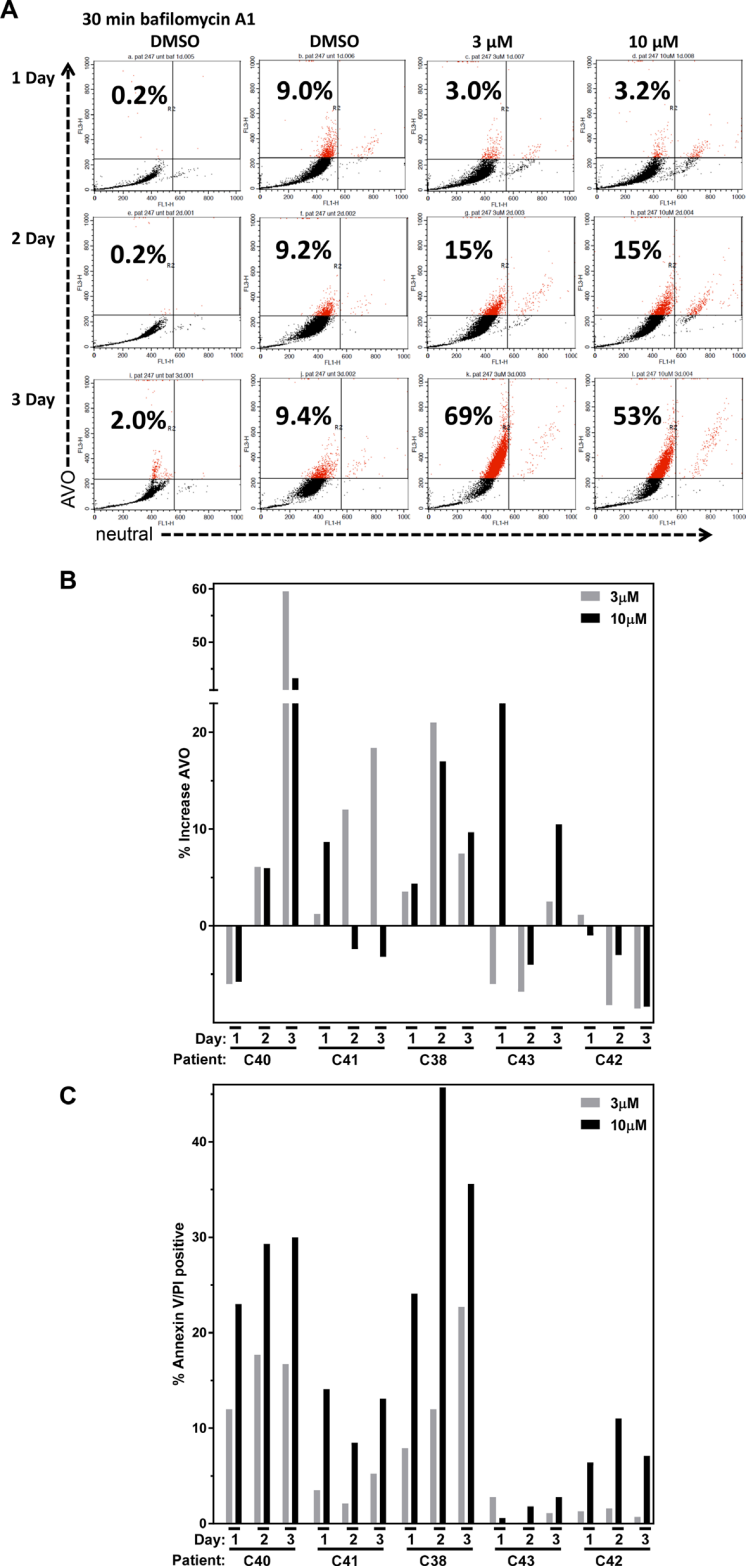
Autophagy and cell death after AZD1208 treatment in chronic lymphocytic leukemia (CLL) cells (**A**) Lymphocytes were isolated from CLL patient blood sample C40 and were untreated or treated with the vehicle (DMSO 0.1%), 3 µM AZD1208, 10 µM AZD1208, or the negative control bafilomycin A_1_ for 24 h. Cells were harvested and analyzed for acidic vesicular organelle (AVO) formation using acridine orange staining and flow cytometry. The number in the upper left corner represents % AVO by acridine orange staining. (**B**) Effect of dose-dependent and time-dependent AZD1208 treatment on %AVO formation and (**C**) % cell death. A total of 5 CLL patient samples were treated as described above and analyzed for AVO formation in parallel for cell death by Annexin V/PI staining assessed by flow cytometry.

Numerous therapeutic modalities have been shown to induce autophagy which has been found to promote survival and thus therapeutic resistance [28]. Because AZD1208 affected 4E-BP1 phosphorylation (Figure 3D) and produced limited cell death in CLL lymphocytes (Figure 1D), we investigated whether AZD1208 induced autophagy in the CLL samples. Autophagy is an intracellular degradation process characterized by acidic vesicular organelle (AVO) formation [29]. Acridine orange dye accumulates in AVOs and can be used to detect AVO formation. CLL cells were treated with DMSO or AZD1208 (Figure 4A). Bafilomycin A_1_ was used as negative control. A time- and dose-dependent increase in AVO staining was observed upon AZD1208 treatment at the indicated concentrations and time points. Based on these preliminary results observed in one patient sample, similar acridine orange staining experiments were performed with four additional CLL patient samples (Figure 4B). There were heterogeneous results from the five different patient samples, though overall most samples resulted in an increase in AVO staining with AZD1208. Cell death by Annexin V/PI positive staining was also performed on the five patient samples (Figure 4C) in parallel to the acridine orange staining experiments to determine the relationship between cell death and autophagy. Endogenous cell death in time matched untreated samples (median, 18%; range, 4%–40%) was subtracted from each treated CLL sample to compare the induction of cell death by AZD1208 treatment. All samples showed a time- and dose-dependent increase in cell death upon AZD1208 treatment.

**Figure 5:**
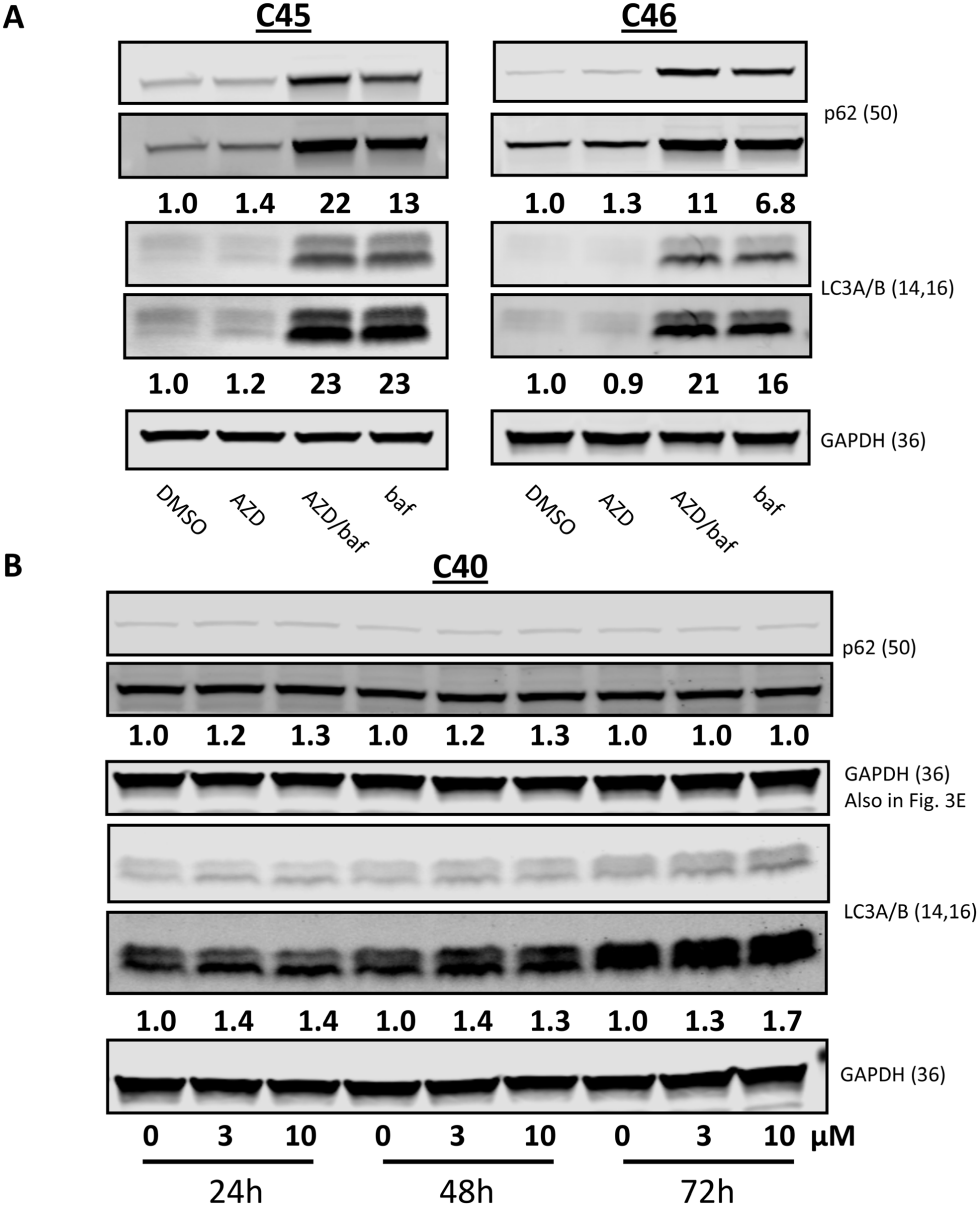
Autophagic markers after AZD1208 treatment in chronic lymphocytic leukemia (CLL) cells (**A**) Effect of AZD1208 treatment on p62 and LC3A/B protein expression. Cells were treated with dimethyl sulfoxide (DMSO) or 10 μM AZD1208, 10 μM AZD1208 plus 0.16 µM bafilomycin A_1_, or 0.16 µM bafilomycin A_1_ for 24 h for samples C45 and C46; cells were then harvested, lysed, and analyzed via immunoblot. Both high and low exposures of the immunoblots are shown. (**B**) Cells from sample C40 were treated with DMSO, 3 µM or 10 µM AZD1208 for 24 h, 48 h, and 72 h followed by immunoblot analysis. Ratios of p62-to-GAPDH or lipidated (LC3A/B-II) to GAPDH were calculated, and the numbers are depicted below the protein bands. Both high and low exposures of the immunoblots are shown. When more than two proteins with the same molecular weight needed to be detected, the membrane was gently stripped and re-probed. In such cases, loading control remains same. Note: GAPDH loading control in Figures 3E and 5B is the same.

Since the five CLL samples stained with acridine orange resulted in different levels of AVO staining after AZD1208 treatment, immunoblots assessing for p62 and LC3A/B were performed (Figure 5A–5B). The p62 protein is an ubiquitin binding protein involved in autophagy and other cellular processes. Decrease in p62 which may be preceded by p62 accumulation in the cell is indicative of an induction of autophagy coupled with increased delivery to, and degradation within autolysosomes [30]. Moreover, increased transcription of p62 upon autophagy induction has also been documented in some cell types including leukemia [31]. LC3 is also an autophagic marker which is cleaved (LC3-I) and lipidated to form LC3-II and is bound to autophagosomes. Because LC3 conversion (LC3-I to LC3-II) correlates with autophagosome formation, LC3-II is another protein used to monitor autophagy [32]. Inhibition of autolysosomal degradation of LC3-II and p62 with agents to inhibit organelle’s proteolytic activity allows for the measurement of autophagic flux as this reveals the amount and rate of cargo sequestering and degradation. Additionally, this type of analysis clarifies that changes in autophagy markers are due to induction of the autophagic process. Two CLL samples were treated with the vehicle, 10 µM AZD1208, 10 µM AZD1208 plus 0.16 µM bafilomycin A_1_, or 0.16 µM bafilomycin A_1_ for 24 h and analyzed for p62 and GAPDH levels (Figure 5A). There is an increase in p62 protein levels with AZD1208 which was greatly augmented by cotreatment with bafilomycin A_1_, demonstrating autophagic flux. CLL sample treated with AZD1208 without bafilomycin A_1_ also showed an increase in p62 level after 24 and 48 hr indicating a fluctuation in these cells (Figure 5B).

The lipidation status of LC3A/B was also assessed by immunoblots under the same AZD1208 treatments (Figure 5A and 5B) using the same CLL samples as describe above for p62 protein levels. Similarly, there was an AZD1208-dependent increase in LC3A/B-II levels which was also greatly augmented by cotreatment of bafilomycin A_1_, again demonstrating autophagic flux. Time course-treated CLL cells showed an increase in LC3A/B-II level at 24, 48, and 72 hr of AZD1208 exposure further indicating that increases in autophagy were sustained during the three days of treatment.

## DISCUSSION

AZD1208 is among the first PIM kinase inhibitors that was being tested in the clinic [21]. Hematological malignancies provide a strong rationale to test PIM kinase inhibitors as PIM enzymes are prevalent in these neoplasms [6–12, 21, 23–26, 33]. Compared to normal lymphocytes, CLL cells also showed higher levels of PIM2 at both transcript and protein levels (Supplementary Figure 1A–1B) [33].

We evaluated the cytotoxic and molecular effect of AZD1208 in lymphocytes isolated from patients with primary CLL. Our data show that AZD1208 induces moderate cell death in CLL cells (6% at 3 µM and 18% at 10 µM; after subtracting endogenous cell death; Figure 1D) and insignificant cell death in healthy lymphocytes (Figure 1B–1C). Activity of AZD1208 needs to be tested in murine models. For example, AML primary cells treated with another PIM kinase inhibitor (SGI-1776) also showed limited cytotoxicity (25% median cell death at 10 µM), but SGI-1776 caused complete regression of the AML tumors in MV-4-11 xenografts [23].

Our *in vitro* model does not take into consideration the antiproliferative effect exerted by PIM kinase inhibitors. Recent reports have challenged the long-standing belief that CLL is a quiescent malignancy driven primarily by the overexpression of antiapoptotic proteins. Evidence now suggests that the bone marrow, spleen, and lymph node niches provide an environment where CLL cells proliferate [34]. The cells used in our study were obtained from blood (nonproliferative); therefore, our model does not assess the ability of AZD1208 to hinder proliferation. In addition, MEC-1 cell line (doubling time about 24 h) when treated with AZD1208 showed only a slight decrease in proliferation. Due to the *in vitro* limitations of our model, animal models should be used to address the anti-proliferative effect of AZD1208.

PIM kinases have been evaluated in the last few years as a therapeutic target for hematological malignancies and solid tumors due to their abundance and constitutive activation in these malignancies [2]. These kinases phosphorylate and regulate a number of protein substrates that play key roles in cell-cycle regulation, transcription, translation, cell survival, drug resistance and inflammation [2, 23]. With regards specifically to CLL, PIM1 has been reported to play an important role in CLL survival though microenvironment signaling via receptor CXCR4 [33]. There was a correlation between CXCR4 phosphorylation and PIM1 protein and transcript levels in CLL cells, and PIM inhibition with small molecules reduced CXCR4 Ser339 phosphorylation and blocked its function. In addition, PIM2 was reported to be overexpressed in poor prognosis CLL cases [35]. A number of PIM inhibitors have been investigated in leukemia cells, [36–39], however, few have been investigated in CLL, thus we evaluated the effect of inhibition of PIM kinases alone using AZD1208.

We tested several PIM kinase substrates to evaluate changes at the molecular level. We first analyzed the transcription regulators histone H3 and c-MYC because we found heterogeneous global transcription inhibition in AZD1208-treated CLL samples (Figure 3A and 3C). Our results show an increase in phosphorylated c-MYC Ser62 after AZD1208 treatment for 24 h, which led to the stabilization of this protein and the promotion of transcription activation. However, *MCL-1* is one of the c-MYC target genes, and our immunoblot analysis results show that MCL-1 levels did not significantly change in our CLL samples (Supplementary Figure 4A and 4B). The effect on MCL-1 protein levels are in contrast to the changes observed with SGI-1776 [7] however, previous studies in malignant B-cell lines showed that impact on MCL-1 protein levels is more pronounced when both Pim kinase 1 and 2 are knocked down [24]. Further analysis is necessary to elucidate the consequence of this increase in phosphorylated c-MYC Ser62 levels. We also performed normalization analyses to compare total histone H3 versus phosphorylated H3 Ser10 levels, and only one CLL sample had decreased phosphorylation status. Considering all these data, AZD1208 does not seem to induce a significant effect in transcription inhibition. PIM kinase cell-cycle substrates were not evaluated in this study because peripheral blood CLL cells are replicationally quiescent. However, we found that AZD1208 inhibited translation in half of the CLL samples (*n* = 8) (Figure 3B). A recent report suggest that PIM1 controls the translation of MET by regulating the phosphorylation of the translation initiation factor eIF4B on S406 in AML cell lines as well as in primary blasts [40]. Both protein synthesis inhibition data (Figure 3B) and 4E-BP1 phosphorylation results suggest heterogeneity. Genetic knockdown of Pim kinase 1 and 2 resulted in malignant B-cell lines showed variabilities, however, knockdown of both isoforms resulted in pronounced effect [24]. In CLL cells in particular, knockdown of Pim 2 and 3 mitigated survival [33]. Using human cell lines and patient-derived tissues acquired from a phase I trial of AZD1208, one study determined that AZD1208 decreased protein synthesis by blocking eIF4B Ser406 phosphorylation by PIM1 [21]. In addition, PIM kinase inhibition by small molecules has also resulted in decreased 4E-BP1 phosphorylation levels in myeloma [41]. Our findings support these reports of the effect of PIM kinase inhibitors on the translation machinery of cancer cells; AZD1208-treated CLL cells showed a decrease in phosphorylated 4E-BP1 Thr37/46 but not in p706SK or phospho-4E-BP1 Ser65 (Figure 3D–3E). Our findings of decreased phosphorylation of the translation repressor 4E-BP1 and decreased protein synthesis, coupled with the lack of significant induction of cell death by AZD1208 (Supplementary Figure 4A–4B), suggest that the primary mechanism of AZD1208 may be the inhibition of protein synthesis. AZD1897, a pan-PIM kinase inhibitor, inhibited the mammalian target of rapamycin (mTOR) pathway in AML cells [42].

Among protein translation inhibitors, mTOR antagonists block cap-dependent protein translation and downstream signaling pathways [43], which in turn initiates the release or binding of the initiation factor eIF4e through 4E-BP1 phosphorylation regulation. A biological consequence of inhibiting mTOR is induction of autophagy.

Therefore, we postulated that AZD1208 could initiate autophagy in a similar way as the blockage of mTOR/4E-BP1 pathway induces apoptosis. We evaluated markers for autophagy as a survival mechanism elicited in cells under stress. Immunoblot results for p62 resulted in accumulation of this protein (Figure 5A) while LC3A/B showed an increase in the lipidated form (LC3A/B-II) in the CLL samples (Figure 5B). In concert, acridine orange staining of AZD1208-treated CLL samples showed increased AVOs in a time- and dose-dependent manner (Figure 4A–4B). These data suggest that AZD1208 treatment results in minimal cell death (Figure 4C), with a concomitant induction of autophagy (Figure 4B) thought the degree of autophagy did not correlate the levels of apoptosis.

Autophagy has been found to promote survival during the stress of chemotherapy, radiotherapy, and targeted therapy resulting in therapeutic resistance [28]. In leukemia, including CLL, it is induced by treatment with various chemotherapeutic drugs. DNA damaging agents have been shown to upregulate the autophagy initiating factor ULK1 in a p53 dependent manner [44]. Fludarabine, CAL-101 (idelalisib), flavopiridol, and ABT-737 have all been shown to induce autophagy [45, 46]. In CLL cells, the histone deacetylase inhibitor, MGCD0103, inhibited autophagy by altering the expression of autophagy genes as well as induced apoptosis [47].

AZD1208 has been found to effectively treat AML by inhibiting cell growth without inducing cell death of healthy cells [21, 40]. This PIM kinase inhibitor was found to suppress translation by regulating the phosphorylation of eIF4B or 4E-BP1 and p70S6K. SGI-1776 has been evaluated in cell lines and patient samples from several hematological malignancies such as CLL [7], AML [23], MCL [24], and myeloma [26]. Though the effect of SGI-1776 on protein translation was not evaluated in CLL, SGI-1776 did not induce significant levels of cell death (only 32% increase in cells treated with 10-µM SGI-1776) [7]. Similarly, low cell death was observed in primary samples of MCL, AML, and MM [23, 24, 26]. However, treatment with SGI-1776 inhibited growth (for MCL and AML), induced autophagy (for MM), and inhibited translation by negatively regulating phosphorylation of 4E-BP1.

To address the lack of cell death induction by AZD1208 and SGI-1776, drug combination strategies have been evaluated using BCL-2 antagonists [48]. Though the data suggested that combination of these PIM kinase inhibitors with two BCL-2 antagonists resulted in additive cytotoxicity with a few synergistic responses, evaluation of other mechanism-based drug combinations could identify synergistic combination strategies.

Recently, the PIM kinase inhibitor LGB321 developed by Novartis was screened against a panel of cell lines from hematological malignancies. This inhibitor was most broadly effective in MM, with some sensitivity in leukemia and lymphoma cell lines; however, the mechanism for each malignancy remains to be elucidated [49]. Several PIM kinase inhibitors were highly active in AML lines and blasts that overexpressed CD25 and in myeloproliferative lines. Importantly, these inhibitors dissipated MYC protein levels which may be due to the protein synthesis effect [37, 50].

In summary, our data suggest that one component of AZD1208 cytotoxicity in CLL cells is through the inhibition of protein translation. Additionally treatment with AZD1208 leads to autophagy in primary malignant lymphocytes. Further research using animal models is necessary to confirm our *in vitro* observations as well as clinical studies to test the efficacy of AZD1208 in treating patients with CLL. Future research could develop combination therapies with AZD1208 that targets autophagy.

## MATERIALS AND METHODS

### Drug

AZD1208 was provided by AstraZeneca Pharmaceuticals LP (Waltham, MA, USA). The drug was dissolved in dimethyl sulfoxide (DMSO) to make 3-mM and 10-mM stock solutions, which were stored at –20°C. All experiments that included a vehicle control were conducted using 0.1% DMSO.

### Patient samples and lymphocyte isolation

We used two protocols to obtain peripheral blood samples from patients with CLL and from healthy donors. All participants signed an informed consent form, and the protocols were approved by The University of Texas MD Anderson Cancer Center institutional review board. Samples were collected in heparinized tubes and diluted in phosphate-buffered saline (PBS). Peripheral blood mononuclear cells (PBMCs) were isolated using the standard Ficoll-Hypaque (Life Technologies, Carlsbad, CA, USA) density gradient centrifugation, as described previously [7]. The isolated PBMCs were washed twice and then were suspended in Roswell Park Memorial Institute (RPMI) 1640 medium supplemented with 10% human AB serum (Cambrex Biosciences, East Rutherford, NJ, USA) with 5% carbon dioxide at 37°C and maintained at 10^7^ cells/mL. Cell number and cell size were determined by a Coulter channelyzer (Beckman Coulter; Brea, CA, USA).

### Cell line

The MEC-1 cell line, derived from a patient with B-chronic lymphocytic leukemia, was a gift from Dr. George Calin at The University of Texas MD Anderson Cancer Center. The cells were maintained in RPMI 1640 medium supplemented with 10% FBS serum (Life Technologies) with 5% carbon dioxide at 37°C and maintained at 10^6^ cells/mL. The cell line was authenticated and was routinely checked for Mycoplasma.

### Clinical laboratory end points

Immunoglobulin heavy-chain variable-region gene mutation status was obtained from MD Anderson Clinic Station and zeta-chain-associated protein kinase (ZAP-70) analysis for the patients in our study was provided by the Chronic Lymphocytic Leukemia Research Consortium (University of California San Diego, San Diego, CA, USA [51]. Fluorescent *in situ* hybridization (FISH) analysis data were provided by the clinical cytogenetics laboratory in the Department of Hematopathology at MD Anderson Cancer Center. The FISH technique was used to detect the chromosome 17p deletion for the p53 gene in CLL cells; this assay has been described previously [52]. Chromosomal cytogenetics was determined using CLL lymphocytes at MD Anderson Cancer Center.

### RNA and protein synthesis assay

Global RNA synthesis and protein synthesis were measured using [^3^H]uridine or [^3^H]leucine incorporation (43 or 5 Ci/mmol. Moravek Biochemicals; Brea, CA, USA), respectively. CLL lymphocytes were treated with either DMSO or indicated concentrations of AZD1208, and 1 h before the end of incubation, cells were labeled with radioactive material at 37°C. The labeled cells were harvested, were washed with ice-cold (4°C) PBS, and were transferred to glass fiber filters (Whatman; Clifton, NJ, USA) using a Millipore vacuum manifold (Thermo Fisher Scientific; Waltham, MA, USA). The filters were then washed in ice-cold (4°C) 0.4 N perchloric acid and were rinsed once with 70% ethanol and dried overnight. The dried filters were transferred to scintillation vials containing high-flashpoint cocktail scintillation fluid (7 mL) (Research Products International; Mount Prospect, IL, USA). Data are expressed as a percentage of the untreated control.

### Apoptosis assay

Cells were untreated or treated with DMSO or AZD1208 and cell death (Annexin V/propidium iodide (PI) positivity) was measured using a Becton Dickinson FACSCalibur flow cytometer (Franklin Lakes, NJ, USA). Briefly, cells were washed, suspended in Annexin binding buffer (200 μL) (Roche; Indianapolis, IN, USA), mixed with Annexin V solution (5 μL) (BD Pharmingen; San Diego, CA, USA) plus PI (5 μL) (Sigma-Aldrich; St. Louis, MO, USA) and incubated for 15 min in the dark at room temperature. At least 10,000 cells were measured per sample.

### Acridine orange staining

Acidic vesicular organelles were quantified using acridine orange dye. Aliquots of 10^6^ CLL cells were transferred to 1 mL RPMI 1640 medium without phenol red (Life Technologies) after treatment with and without AZD1208. To an aliquot of untreated cells, 0.1 μg/mL bafilomycin A_1_ (Sigma-Aldrich) was added as a control for negative AVO staining. All cells were incubated for 30 min at 37°C, 5% CO2. The cells were then stained directly in culture with 1 μg/mL acridine orange (Life Technologies) at 37°C, 5% CO2 for 15 min in the dark. Cells were centrifuged (1500 rpm for 5 min) and suspended in 0.5 mL of RPMI 1640 medium without phenol red. Accumulation of acidic vesicular organelles was quantified using the Becton Dickinson FACSCalibur system as described [53].

### Protein extraction and immunoblot assays

Cells were untreated or treated with DMSO or AZD1208. CLL cells were harvested after treatment, centrifuged, and washed twice with PBS. Cells were lysed using one tablet of Complete Mini Protease Inhibitor Cocktail (Roche) in 10 mL of 1 x radioimmunoprecipitation assay buffer (Bio-Rad; Hercules, CA, USA) or 1 x Lysis Buffer (Cell Signaling). The lysate protein content was measured using a DC protein assay kit (Bio-Rad), according to the manufacturer’s instructions. Protein samples were electrophoresed on Criterion XT Bis-Tris gels using either XT MOPS buffer or XT MES buffer (Bio-Rad) and were transferred either to nitrocellulose or PVDF membranes. Primary antibodies were purchased from the following sources: BCL-2 (Dako; Carpinteria, CA, USA); MCL-1, BCL-X_L_, and c-MYC (clone C33) (Santa Cruz Biotechnology; Santa Cruz, CA, USA); phospho-4E-binding protein 1 (4E-BP1) (Thr37/46), phospho-4E-BP1 (Ser65), total 4E-BP1, phospho-p70 S6 kinase (Thr389), total p70 S6 kinase, glyceraldehyde 3-phosphate dehydrogenase, total Bad, total histone H3, and LC3A/B (Cell Signaling Technology; Danvers, MA, USA); phospho c-MYC-1 (Abcam; Cambridge, MA, USA); phospho-histone H3 (Ser 10) (EMD Millipore; Billerica, MA, USA); and poly ADP ribose polymerase (BD Pharmingen); p62 (Enzo Life Sciences, Farmingdale, NY).

### Gene expression assays

CLL lymphocytes were left untreated, or treated with DMSO or AZD1208. Total RNA was isolated using an RNeasy Mini Kit (Qiagen; Valencia, CA, USA) and quantified using the NanoDrop ND 1000 spectrophotometer (Thermo Fisher Scientific). Relative transcript levels of gene expression were assessed using TaqMan One-Step RT-PCR Master Mix reagents (Applied Biosystems; Foster City, CA, USA). Predesigned primers and TaqMan probes for eukaryotic 18S ribosomal RNA (4333760), *PIM1* (Hs01065498_m1), *PIM2* (Hs00179139_m1), *PIM3* (Hs00420511_g1), *BCL-2* (Hs00608023_m1), *BCL-X*_L_ (Hs00236329_m1), and *MCL-1* (HS00172036_m1) were purchased from Applied Biosystems. Relative levels of gene expression were measured with an ABI prism 7900 Sequence Detection System (Applied Biosystems) and determined using standard curves following normalization to the endogenous gene glyceraldehyde 3-phosphate dehydrogenase (GAPDH). Experiments were done in triplicate, and the results were plotted as fold change in comparison with untreated cells.

### Statistical analysis and graphs

We used GraphPad Prism (GraphPad Software, version 5; San Diego, CA, USA) for all plots and analyses as well as to determine *P* values using the Mann-Whitney test (a nonparametric test that compares two unpaired groups). *P* values less than 0.05 were considered statistically significant.

## SUPPLEMENTARY MATERIALS FIGURES



## Abbreviations

PIM: proviral integration site for moloney murine leukemia virus
CLL: chronic lymphocytic leukemia
MCL: mantle cell lymphoma
AML: acute myelogenous leukemia
4E-BP1: 4E-binding protein 1
ZAP-70: zeta-chain-associated protein kinase
DMSO: dimethyl sulfoxide
FBS: fetal bovine serum
PBS: phosphate-buffered saline
PBMC: peripheral blood mononuclear cells
