# Supplementary material for: PIM kinase inhibitor, AZD1208, inhibits protein translation and induces autophagy in primary chronic lymphocytic leukemia cells
Source: Oncotarget. 2019 Apr 19;10(29):2793–809. doi: 10.18632/oncotarget.26876 (PMC6497463; doi:10.18632/oncotarget.26876)
Supplement: Supplementary file 1 [file oncotarget-10-2793-s001.pdf]

PIM kinase inhibitor, AZD1208, inhibits protein translation and induces autophagy in primary chronic lymphocytic leukemia cells

SUPPLEMENTARY MATERIALS

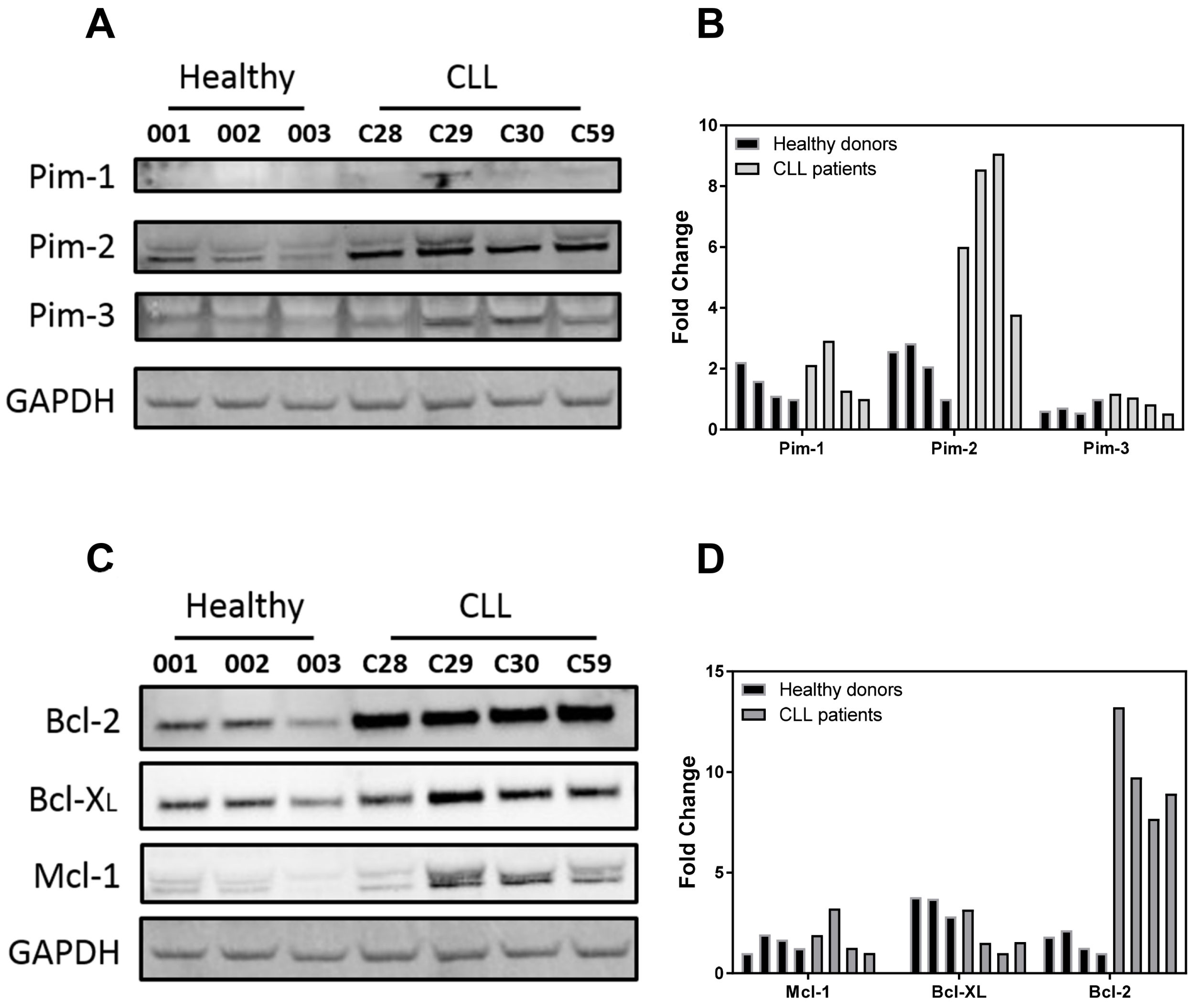

Supplementary Figure 1: Expression of PIM kinase and antiapoptotic proteins and transcript levels in healthy and chronic lymphocytic leukemia (CLL) lymphocytes. (**A**) Protein expression of PIM1, PIM2, and PIM3 in untreated healthy and CLL primary cells. Lymphocytes from healthy donors and CLL patients were lysed and analyzed by immunoblot for PIM kinases with glyceraldehyde 3-phosphate dehydrogenase (GAPDH) as a loading control. (**B**) mRNA levels of PIM1, PIM2, and PIM3 in untreated healthy and CLL primary cells. Lymphocytes from healthy donors and CLL patients were lysed and analyzed by real time reverse transcription polymerase chain reaction for the PIM kinases with eukaryotic 18S ribosomal RNA used as an internal control. (**C**) Protein expression of MCL-1, BCL-X_L, and BCL-2 in untreated healthy and CLL primary cells. Lymphocytes from healthy donors and CLL patients were lysed and analyzed by immunoblot for the three antiapoptotic proteins, with glyceraldehyde 3-phosphate dehydrogenase (GAPDH) as a loading control. (**D**) mRNA levels of MCL-1, BCL-X_L, and BCL-2 in untreated healthy and CLL primary cells. Lymphocytes from healthy donors and CLL patients were lysed and analyzed by real time reverse transcription polymerase chain reaction for the three antiapoptotic proteins, with eukaryotic 18S ribosomal RNA used as an internal control. Note: GAPDH loading control in Supplementary Figure 1A and 1C is the same.

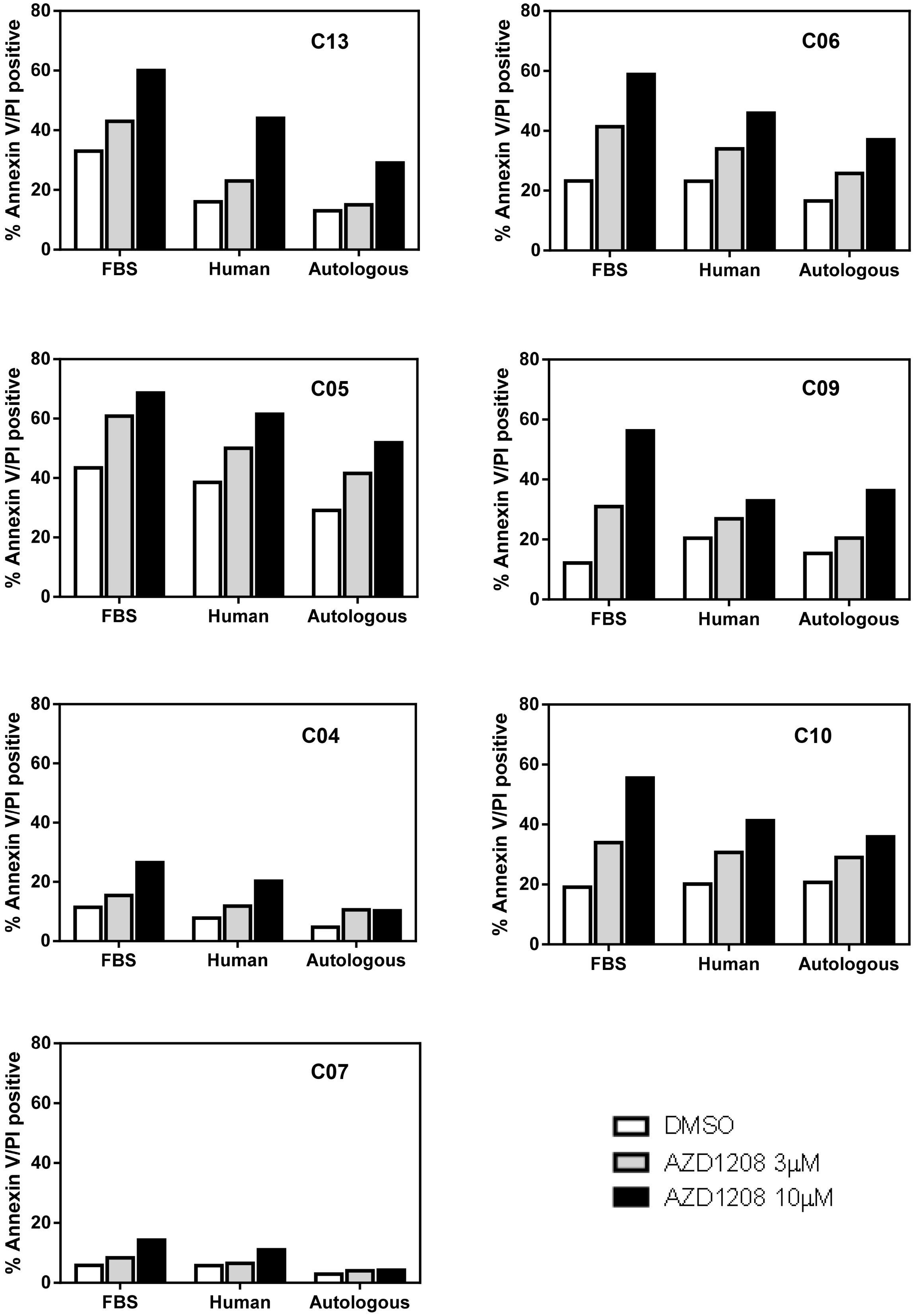

Supplementary Figure 2: Impact of different types of serum on AZD1208-induced apoptosis in chronic lymphocytic leukemia (CLL) cells. CLL lymphocytes from seven CLL patients were collected and cultured in media supplemented with 10% fetal bovine serum (FBS), 10% human serum (human), or 10% autologous serum (autologous). Each sample was treated with either dimethyl sulfoxide (DMSO) (0.1%), 3 µM, or 10 µM AZD1208 for 24 h and cell death was determined using flow cytometry after Annexin V/ propidium iodide (PI) staining.

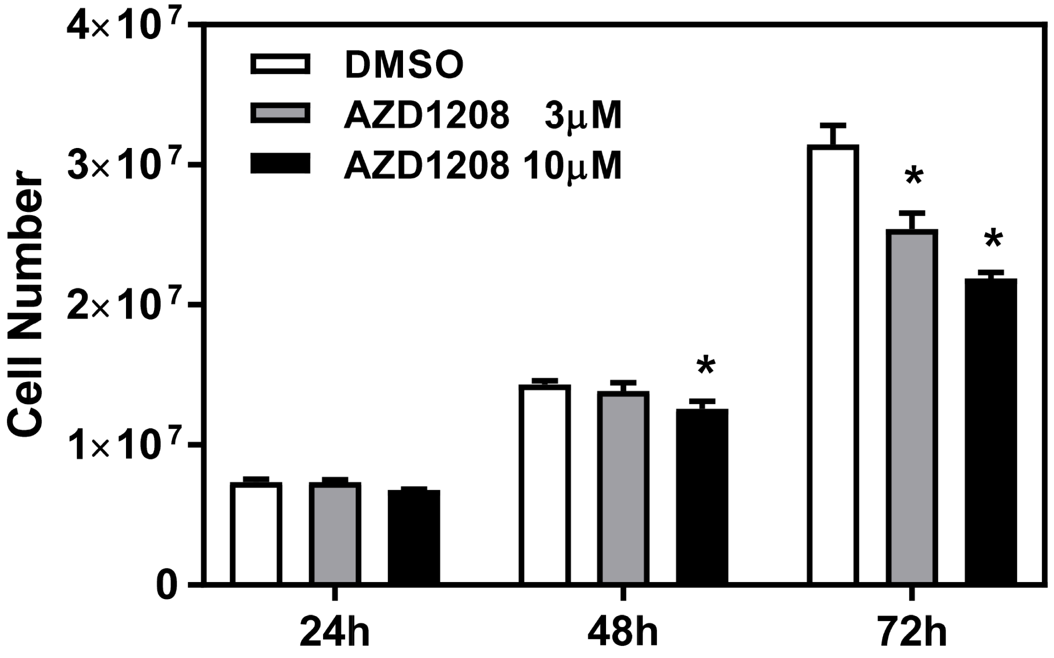

Supplementary Figure 3: Effect of AZD1208 on proliferation in MEC-1 cells. MEC-1 cells were incubated with dimethyl sulfoxide DMSO, 3 μM AZD1208, or 10 μM AZD1208 and cells were counted using a Coulter channelyzer at 24, 48, or 72 h. Experiments were done in triplicate and plotted as the total cell number for each condition at the indicated time points. Error bars represent SEM and bars with an asterisk on the top had *p* value less than 0.05 compared to DMSO value.

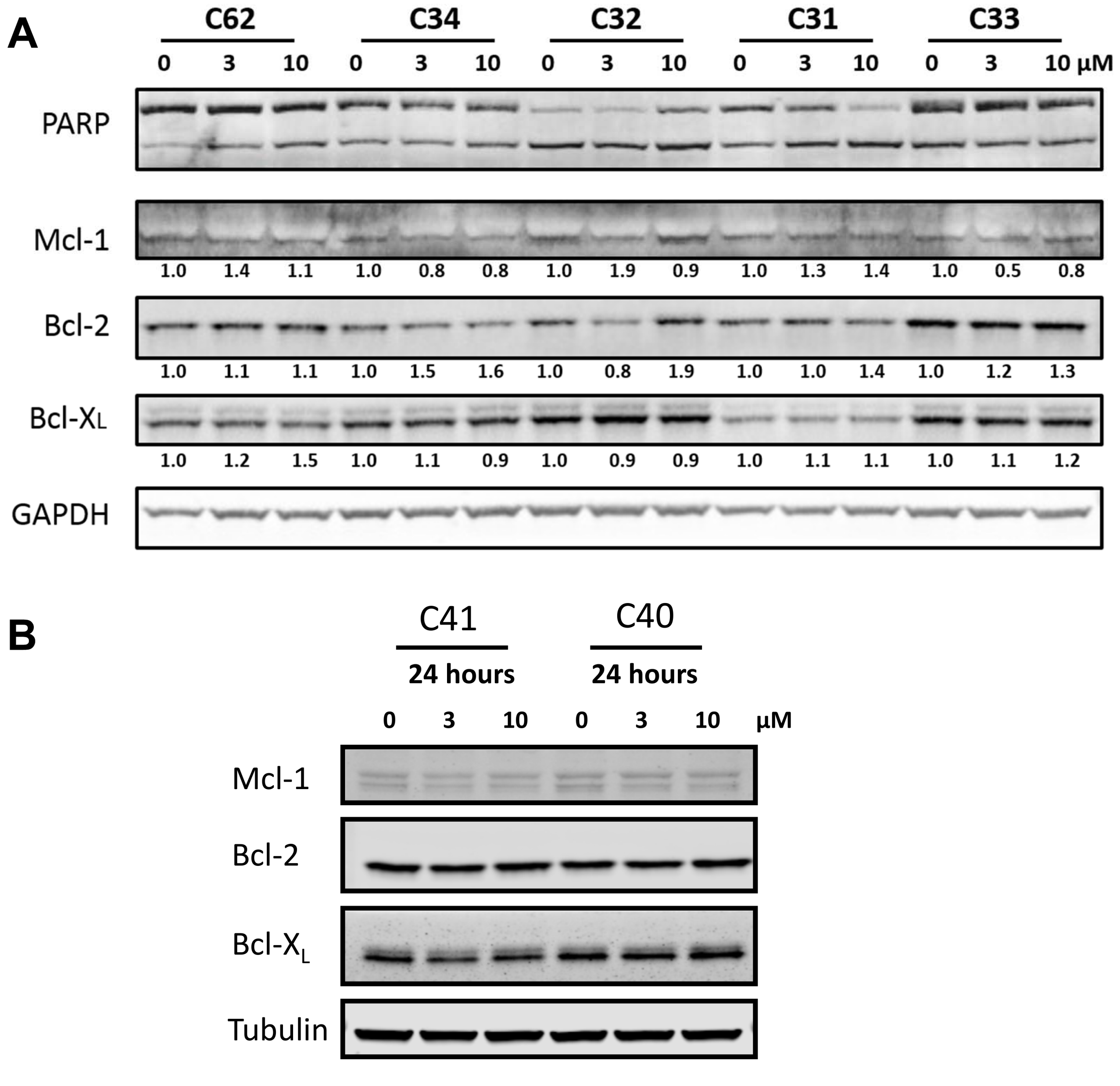

Supplementary Figure 4: Impact of AZD1208 on early response and survival genes in chronic lymphocytic leukemia (CLL) cells. Effect of AZD1208 on PARP cleavage and several BCL-2 family protein expression. Cells were treated with dimethyl sulfoxide (DMSO) or AZD1208 (3 μM or 10 μM) for 24 h; cells were then harvested, lysed, and analyzed via immunoblot. (**A**) Bcl-2 family antiapoptotic proteins were probed from 5 patient samples and (**B**) additional two patients. Total-to- GAPDH protein ratios were calculated, and the numbers are depicted below the protein bands. The membrane was probed with LI-COR imaging system using anti-rabbit and anti-mouse with distinct fluorescent wavelengths. When more than two proteins with the same molecular weight needed to be detected, the membrane was gently stripped and re-probed. In such cases, loading control remains same. Note: GAPDH loading control for Figure 3C, 3D (bottom gel), and Supplementary Figure 4A is the same.
